# Understanding Thoracic Outlet Syndrome

**DOI:** 10.1155/2014/248163

**Published:** 2014-07-20

**Authors:** Julie Freischlag, Kristine Orion

**Affiliations:** ^1^Division of Vascular Surgery, Department of Surgery, The Johns Hopkins Hospital, 720 Rutland Avenue, Ross 759, Baltimore, MD 21205, USA; ^2^Division of Vascular Surgery, Department of Surgery, The Johns Hopkins Hospital, 600 N. Wolfe Street, Halsted 668, Baltimore, MD 21287, USA

## Abstract

The diagnosis of thoracic outlet syndrome was once debated in the world of vascular surgery. Today, it is more understood and surprisingly less infrequent than once thought. Thoracic outlet syndrome (TOS) is composed of three types: neurogenic, venous, and arterial. Each type is in distinction to the others when considering patient presentation and diagnosis. Remarkable advances have been made in surgical approach, physical therapy, and rehabilitation of these patients. Dedicated centers of excellence with multidisciplinary teams have been developed and continue to lead the way in future research.

## 1. Introduction

Thoracic outlet syndrome (TOS) is a disease which involves compression of the neurovascular bundle as it exits the thoracic girdle. Twenty years ago, the existence of such a syndrome was poorly understood and therefore its acceptance as a medical diagnosis was controversial [1]. Today, however, the syndrome is better recognized; its symptoms and pathophysiology are more clearly defined. And, as the number of patients undergoing successful treatment increases, TOS is becoming a common diagnosis in vascular surgery clinics around the world.

## 2. Embryology

**Figure 1:**
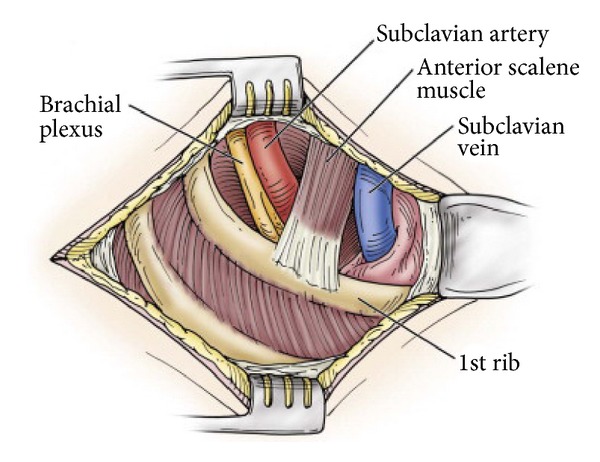
Anatomy of the thoracic outlet (reprinted with permission of Elsevier; see [8]).

The thoracic outlet is anatomically defined by the space between the first thoracic vertebra, first rib, and manubrium of the sternum. Anteriorly, the subclavius tendon lies next to the subclavian vein. Next, the scalene separates the subclavian vein from the subclavian artery. The brachial plexus travels posterior and laterally to the artery and is accompanied by the middle scalene muscle (Figure 1). It is a very small space, already occupied by the anterior scalene, the subclavius, and prevertebral muscles. The thoracic outlet is also dynamic; the volume changes with respiration and any activity of the neck, thorax, or arm [2]. Additionally, cervical ribs or anomalous first ribs, which tend to be more cephalad or fused with the second rib, can further affect the dimensions of the thoracic outlet [3]. As the space continuously contracts and expands, there may be impingement of the brachial plexus or subclavian vessels by the osseous structures. Simultaneously, fibrosis and scarring may occur, which can then cause encroachment or inflammation.

## 3. Pathogenesis and Clinical Presentation

There are three types of TOS: neurogenic, venous, and arterial. As these structures travel together while exiting the thorax and entering the axilla, it can be understood why any of the three can be affected. What is not so understood is why neurogenic is much more common than either the venous or arterial thoracic outlet syndrome.

Neurogenic thoracic outlet syndrome (nTOS) makes up approximately 95% of all patients suffering from this syndrome. Patients can present with vague symptomatology but most often complain of pain and numbness in their fingers, hands, or arms on the affected side. Many of these patients have experienced some type of trauma, typically motor vehicle accidents or falls. Patients seem to additionally describe a history vigorous repetitive activity, likely related to their profession or lifestyle. In our practice, 50% of patients present with a history of either trauma or chronic repetitive motion. It is suspected that the formation of scar tissue and adhesions may play a likely role in this relationship. Tissue contraction from scar or adhesions can certainly affect the dynamics of the already small thoracic outlet. Furthermore, they may directly impinge on the nerves or vessels.

Venous thoracic outlet syndrome (vTOS) makes up an additional 3–5% of patients. This group of patients suffers from compression of the axillosubclavian vein which ultimately results in thrombosis. It has been described as “effort thrombosis” or Paget-Schroetter syndrome [4]. Thrombosis may be acute or chronic, occlusive or partially obstructive. Patients will report acute onset purple-red discoloration of a swollen extremity. It is felt that the vein may most often be inflamed causing scarring and narrowing over time until acute thrombosis. There is usually concurrent dull aching pain especially in the recumbent position as well as a sense of heaviness. As time progresses, patients will notice visibly dilated superficial veins across the shoulder, chest, back, and neck as explained by venous collateral development [5]. vTOS is also associated with trauma or repetitive motion. Patients may have a hypercoagulable history; however, it is rare (5–15%). Occasionally, a medical implant such as central venous catheters, pacemakers, or defibrillator wires may be present and, therefore, those patients do not have compression as their etiology for venous thrombosis, but a foreign body.

**Figure 2:**
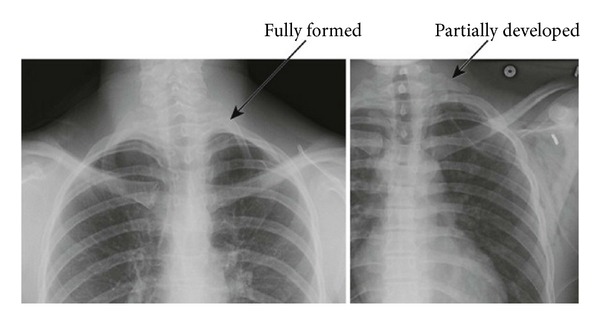
Radiographs show both a fully formed and a partially developed cervical rib (reprinted with permission of Elsevier; see [8]).

Finally, arterial thoracic outlet syndrome (aTOS) is the rarest form accounting for only 1-2%. Continuous friction of the subclavian artery and the underlying first rib from pulsation and activity can cause fibrosis and stenosis of the artery. Poststenotic aneurysms can then form. Both the narrowed artery and aneurysms can cause arterial thrombosis. In the chronic phase, patients experience claudication or pain with activity of the arm/hand which generally subsides with the cessation of movement. In the subacute stage, as emboli form and travel distally, patients present with focal symptoms of ischemia in the form of a severely painful blue or white finger [6]. Acutely, the subclavian artery can completely thrombose, leaving the patient with a threatened limb. Many of these patients will have a cervical rib or other rib anomaly such as a rudimentary rib or fused cervical, 1st and/or second ribs (Figure 2). A recent study from Michigan found an astonishing incidence of 29% for bony abnormalities in their TOS patients and the strongest association occurred with arterial TOS [7].

## 4. Diagnosis

TOS is often a diagnosis of exclusion. Other causes such as herniated cervical disks, rotator cuff injuries, peripheral nerve entrapment, chronic pain syndromes, psychological conditions, multiple sclerosis, hypercoagulable disorders, atrial fibrillation with distal emboli, and upper extremity deep venous thrombosis all need to be considered because they can mimic the symptoms of TOS [9].

Venous and arterial TOS are more easily diagnosed with a combination of clinical presentation and imaging. In vTOS, a duplex ultrasound is the most dominant noninvasive study. A complete evaluation of both the subclavian and axillary veins is important using both grey-scale imaging and Doppler spectral waveform analysis. Velocities and waveforms are obtained in resting and the abducted position of the arm. Dampening of the waveform or marked decrease in velocities while the arm is abducted can assure the diagnosis of vTOS. Patients will frequently have already been diagnosed with some type of venous thrombosis which can also be seen on ultrasound.

Using ultrasound for diagnosis of arterial TOS is similar. Arterial pulse waveforms are again reduced; however, velocities will be increased with stenosis or absent in complete occlusion. Most patients should have a plain chest X-ray to evaluate for cervical ribs or other bony abnormalities. Finally, CTA or traditional angiography can be utilized to identify more clearly the occlusion, aneurysm, or distal embolization.

Diagnosing neurogenic TOS is the least clear. It is not uncommon for patients to have been diagnosed with several other conditions and have failed their treatment plans such as cervical fusion, carpel tunnel release, surgical repair of the rotator cuff, or simply prolonged physical therapy. Intuitively, if the brachial plexus is impinged upon and plexopathy occurs, an MRI should show some type of inflammation of the nerve or surrounding soft tissue, but this has not been consistently true.

Several maneuvers have been developed which together with a good clinical history may diagnose nTOS. The elevated arm stress test (EAST) is a quick assessment in which the patient is asked to abduct the shoulder 90 degrees, flex the elbows at 90 degrees, and face the hands forward while alternately opening and closing their hands at a rate of one per second for 3 minutes. One can actually see decreased ability to open and close their hands or lowering of the arm during the EAST in patients with neurogenic TOS. Patients rate the amount and location of discomfort, pain, fatigue, and numbness. Unfortunately, those with carpal tunnel syndrome, ulnar neuropathy, or fibromyalgia will demonstrate symptoms as well. The Adson maneuver may be performed seated or standing. The patient is requested to take a deep breath and rotate and extend their head as far as possible towards the unaffected side. The affected side arm is abducted with the elbow flexed, and the examiner's fingers should be placed on the radial pulse. The test will reproduce symptoms or obliterate the ipsilateral radial pulse. One can also listen for a bruit underneath the clavicle during the Adson's test to document compression. Another positive finding suggestive of nTOS is tenderness to palpation at the scalene triangle or the insertion of the pectoralis minor.

A more objective examination is the lidocaine scalene block test. Under image guidance, either computed tomography, ultrasound, or fluoroscopy, the anterior scalene muscle is injected with lidocaine. Patients with nTOS should have some decrease or complete relief of symptoms for four hours. In our practice, an initial lidocaine block is utilized and, if positive, predicts 90% success for subsequent treatments including physical therapy and surgical intervention. Interestingly, our review of 159 patients showed that younger patients (<40 years) are more likely to achieve symptom relief overall after transaxillary decompression; however, successful lidocaine blocks better predicted increased surgical success in patients >40 years old [10].

Additionally, botulinum toxin A (Botox) can be used for temporary symptom respite. We frequently perform this injection at a second setting. Botox takes two weeks to work but can last three months and can help patients progress with physical therapy. This technique has been examined nationally, including a study from Boston which concluded that botulinum toxin injection with ultrasound guidance is safe and well tolerated in subjects with suspected nTOS for interim relief [11].

In our institution, we begin our diagnosis algorithm with a good history and physical as well as vascular lab studies if arterial or venous outlet syndrome is suspected. If our vascular lab duplex is consistent with vTOS or aTOS, we will schedule the patient for surgery (see next paragraph). We do not routinely utilize venous lysis preoperatively, nor do we employ CT venogram until the postoperative time period. It is useful to perform CTA in aTOS, especially when arterial reconstruction is considered. In neurogenic TOS, most patients will already come with chest X-rays and cervical spine MRIs. We usually do not require any more imaging. We perform several clinical maneuvers as previously mentioned, and if we suspect nTOS, we refer the patient to rehab. Many of our patients will resolve with physical therapy. Of those still symptomatic and compliant to the physical therapy regimen, may then be scheduled for a lidocaine scalene block test. If the block provides a decrease or relief of symptoms, we will evaluate the patient's overall fitness for surgery.

## 5. Treatment

Surgical intervention, specifically first rib resection and anterior scalenectomy (FRRS), is indicated for both venous and arterial TOS. Sequelae of both types are bound to recur without a definitive operation. Traditionally, anticoagulation and arm elevation was the treatment for vTOS. In more recent years, lysis with or without venoplasty has been offered for patients with vTOS. They were then referred to vascular or thoracic surgeons for a first rib resection three months later. However, we know now that preoperative thrombolysis and venoplasty impart no benefit over routine anticoagulation in regard to vein patency or symptom resolution for these patients. In fact, many will rethrombose in the 3-month interval, awaiting surgery [12]. We recommend FRRS (described in the following section) once the patient has been diagnosed with vTOS. Patients will be at various stages in treatment, some having undergone lysis or plasty and some not having. Anticoagulation is then resumed three days after surgery. If the patient has undergone thrombolytic therapy, we try to schedule them as soon as possible. Invasive venography is performed two weeks postoperatively to identify subclavian vein pathology that may require further venoplasty. Those patients who have lesion-free patent subclavian veins are told to stop anticoagulation. Those undergoing additional endovascular treatment are continued on anticoagulation for 1-2 months until followup. If duplex ultrasound demonstrates vein patency at that time, anticoagulation is stopped. If thrombosis persists, anticoagulation is generally continued for a maximum of 6 months. The need for long-term anticoagulation may vary if the affected patient has an additional underlying hypercoagulable condition.

Uniformly, all patients with arterial thoracic outlet syndrome will need full anticoagulation and varying degrees of surgical intervention. aTOS is the rarest form of this disease and can be quite unique to each patient in regard to symptoms, acuity of presentation, and aneurysmal degeneration. If an aneurysm is present, the patient may require an arterial reconstruction in addition to FRRS. This may alter one from a transaxillary approach to a supraclavicular technique. Arterial reconstruction with an interposition bypass may be achieved using saphenous vein or synthetic grafts.

Treatment for neurogenic TOS, although less clear in the past, is more certain today. Clinicians agree that the initial management is nonoperative. Approximately 60–70% of patients with nTOS can be successfully treated with avoidance of activities that precipitate symptoms, ergonomic modifications to the workplace, and selective use of pharmacologic agents such as nonsteroidal anti-inflammatories, antidepressants, and muscle relaxants. Physical therapy is also a very important component for these patients. Conservative management should be attempted for 8–12 weeks before considering surgery. Those that fail should undergo a lidocaine scalene muscle injection. If they respond to this block, they may be evaluated to see if they are physically fit for FRRS.

## 6. First Rib Resection and Scalenectomy (FRRS)

**Figure 3:**
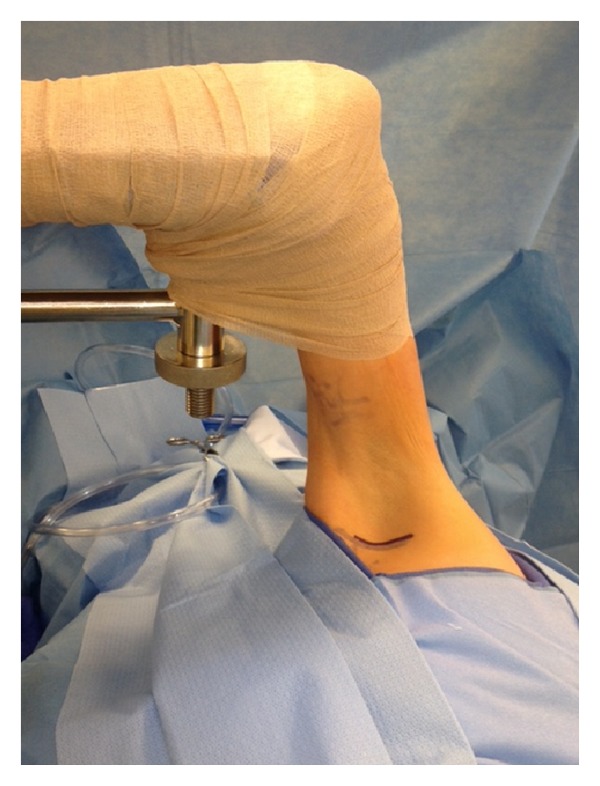
Machleder retractor with incision marked just below axillary hair line.

**Figure 4:**
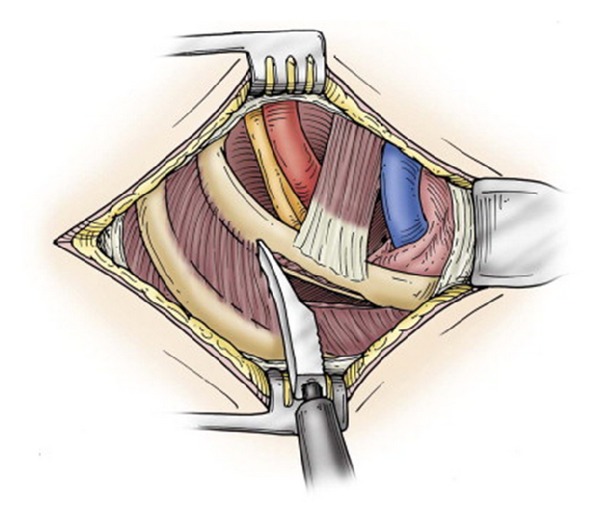
Use of the periosteal elevator to remove tissue from the first rib (reprinted with permission of Elsevier; see [8]).

**Figure 5:**
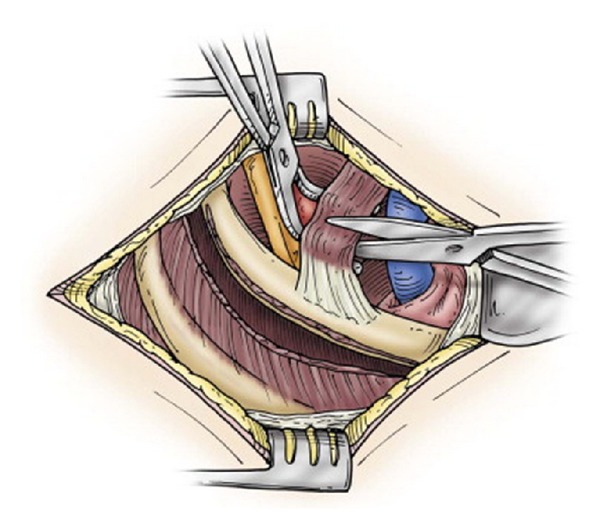
Transection of the anterior scalene muscle with a right angle clamp and scissors (reprinted with permission of Elsevier; see [8]).

**Figure 6:**
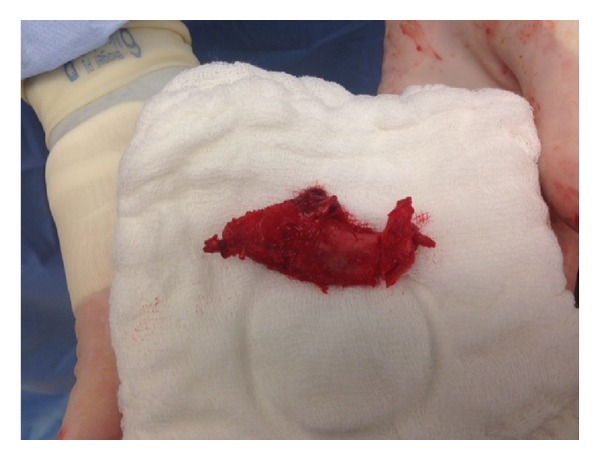
First rib removed.

FRRS has been performed via a supraclavicular incision; however, we favor the exposure of a transaxillary approach and use this technique for all our patients regardless of size. FRRS is performed under general anesthesia with avoidance of any long-acting paralytics for intraoperative nerve identification and monitoring. Positioning and retraction are the most important aspects of obtaining an adequate surgical field of view [13]. The patient is placed on a bean bag in the lateral position with the operative side up. Ample padding with foam or pillows should be used in order to protect pressure points as the bean bag is inflated. The axilla and arm are prepped circumferentially to the wrist and a Machleder retractor is utilized. This retractor allows excellent exposure of the thoracic outlet without the laborious need for an assistant to extend the arm throughout the surgery. The borders of the latissimus dorsi muscle and pectoralis major muscle are marked, and the incision is made between the two just below the axillary hair line (Figure 3). Dissection is taken down directly to the chest wall in order to avoid disturbing the axillary lymphatic bed. Gentle blunt dissection is then performed towards the apex of the axilla and first rib. Lighted hand-held retractors are used to provide illumination deep into the operative field. The anatomy is identified; the vein will be fluttering with respiration; the artery will be pulsatile (in vTOS, the vein can be fibrotic and more difficult to detect). The first rib is identified as the scalene muscle will insert on its most cephalad edge. Sharp periosteal elevators are used to clear intercostals and mobilize the first rib (Figure 4). Inferiorly, the pleura is gently peeled off the rib, but pneumothorax can be encountered in patients with significant scarring. Next, the subclavius muscle is divided sharply, which is followed by high division of the anterior scalene muscle (Figure 5). Great care must be taken to avoid injury to the artery during this step. Once the rib is completely mobilized, a rib cutter is used anteriorly first, next to the subclavian vein. Finally, the rib is then transected posteriorly at the level of or just anterior to the brachial plexus. Frequently a second transection is performed just behind the brachial plexus to assure that no scarring will occur between the nerves and the rib in the postoperative period causing recurrent symptoms 20% of the time. The rib is removed (Figure 6) and the space is inspected for hemostasis. If a pneumothorax has occurred, a small 12-French chest tube is placed prior to closing the incision in two or three layers.

Postoperatively, the patient is kept overnight for observation after a plain chest radiograph is performed in the recovery area. Pain is controlled with a PCA and muscle relaxers. The patient is provided with a sling for comfort and is allowed immediate ambulation. Chest tubes are removed in the morning, and 95% of patients are discharged home the following day.

All patients receive physical therapy starting 2 weeks after the surgery. Physical therapy is a specific 8-week regimen that focuses on postural training as well as strength training in the shoulder, chest, and neck. During weeks 1–3, therapists work on mobilizing the thoracic and cervical spine using thoracic extension and cervical flexion. This also begins to tease the patient's stretch and pain sensations. Patients concentrate on diaphragmatic breathing and perform gentle scapular posterior depression exercises. Finally, they start posture training. In Weeks 4–6, patients add mild resistance training to improve scapular stability and mobility of the glenohumeral joint and rotator cuff.

The most common complication for FRRS is pneumothorax and occurs up to 10% of the time. Bleeding and need for reoperation are rare but we still avoid using strong nonsteroidals such as Toradol in the immediate postoperative setting. Infection is also uncommon despite the proximity of the incision to the axillary hair line and apocrine sweat glands. We utilize skin glue on the incision which aids in preventing contamination with sweat. Injuries to the brachial plexus or long thoracic nerves occur in <1% of cases.

## 7. Outcomes after FRRS

Neurogenic TOS is the most investigated form of TOS. Because surgical treatment focuses on alleviation of pain and numbness, outcomes must be analyzed using subjective means. Several forms can be utilized: Short-Form 12 (SF-12), Brief Pain Inventory (BPI), and Cervical Brachial Symptom Questionnaire (CBSQ). Previous studies have shown improvement in quality of life with short-term followup (2 years after FRRS) but then a decline at three years. A more recent report including 87 nTOS patients demonstrated no difference in quality of life between short-term followup and a prolonged followup of 44 months. Overall, 90% of patients will have relief of their symptoms after FRRS [14].

Therefore, symptom recurrence or surgical failure happens 10% of the time. As already mentioned, a positive lidocaine scalene block is a clinical indicator that a patient will have a successful response to surgical intervention but this is not a guarantee. In our analysis of 182 patients at the Johns Hopkins Hospital [15] having undergone FRRS, older age (>40) was significantly predictive of a poor outcome. Longer duration of symptoms and active smoking were more associated with unresolved symptoms. Finally, those patients with comorbid chronic pain syndromes are more likely to have persistent pain postoperatively. What is interesting is that, in contrast to unresolved symptoms, patients with recurrent symptoms are well managed with physical therapy. Additionally, Botox injections can be helpful with recurrent symptoms to relax the painful muscles in the shoulder and back areas. All surgical failures or recurrences for nTOS should also be reevaluated in clinic for contralateral symptoms. Bilateral nTOS can occur frequently in patients with cervical ribs. A second FRRS after 1 year may improve their results. We wait a year to ensure adequate recuperation from the first operation.

In 2008, we looked at the rate of return to work for our TOS patients. Although 34% of neurogenic and 8% of venous patients were disabled or unemployed upon presentation, astounding 50% of neurogenic and 77% of venous patients eventually returned to activity or full-time work over a 12-month followup [16].

## 8. Recent Developments in TOS

As more and more patients are treated for TOS, the referral pattern has begun to change. Typically, patients were evaluated and treated by several other disciplines before being considered for TOS. Now, patients are being referred earlier with a shorter duration of symptoms, which improves their chance of a successful surgical treatment. A rise of presentation in adolescents has also been observed. Case reports have emphasized that these younger patients have more prevalent unusual anatomy such as cervical ribs or abnormal tendon insertions. Many adolescents will describe repetitive and vigorous activity such as competitive sports or instrumental performance. Noteworthy, adolescents also seem to present more frequently with venous or arterial TOS than adults [17]. FRRS has been safely and successfully performed in this population with a rapid return to their busy lives.

Modern experience indicates that a multidisciplinary comprehensive approach to TOS improves outcomes. Centers of TOS excellence have been established throughout the country which provide a well-organized and uniformed process for these patients [18]. Dedicated nurses, physician assistants, physical therapists, medical physicians, surgeons, neurologists, and pain management anesthesiologists who understand TOS can efficiently diagnose patients, create focused management algorithms, and deliver specialized postoperative attention.

## 9. Future Research

Although TOS has come a long way in the last half century, there are many avenues left to explore. Diagnosis still remains the most debated aspect of neurogenic TOS. Despite multiple maneuvers and even the lidocaine scalene block, the test results rely on patient symptomatology alone. Continued research would be beneficial to find a more objective analysis. Some have suggested using MRI or CTAs preoperatively to compare TOS patients with control patients to further assist in diagnosis. Secondly, in the case of aTOS, it is unclear if prosthetic or autologous tissue is better for reconstruction. Patency rates and need for reoperations are important aspects as in any vascular reconstruction. As arterial TOS is the rarest form, a study to resolve this issue would require multiple centers. Finally, we have adopted the protocol of performing venography two weeks after first rib resection and scalenectomy. There may still be some amount of postoperative inflammation at this interval, so the timing of these venograms should be investigated.

## 10. Conclusion

Today, we can begin to understand thoracic outlet syndrome. The embryology and pathophysiology make sense. We appreciate that there are three types of thoracic outlet syndrome. Each type has a different patient presentation; yet all three can be successfully treated with FRRS. The controversy of whether thoracic outlet syndrome exists is slowly being put to rest.
